# Interspecific Proteomic Comparisons Reveal Ash Phloem Genes Potentially Involved in Constitutive Resistance to the Emerald Ash Borer

**DOI:** 10.1371/journal.pone.0024863

**Published:** 2011-09-15

**Authors:** Justin G. A. Whitehill, Alexandra Popova-Butler, Kari B. Green-Church, Jennifer L. Koch, Daniel A. Herms, Pierluigi Bonello

**Affiliations:** 1 Department of Plant Pathology, The Ohio State University, Columbus, Ohio, United States of America; 2 Department of Molecular and Cellular Biochemistry, The Ohio State University, Columbus, Ohio, United States of America; 3 Northern Research Station, United States Department of Agriculture (USDA) Forest Service, Delaware, Ohio, United States of America; 4 Department of Entomology, Ohio Agricultural Research and Development Center, The Ohio State University, Wooster, Ohio, United States of America; Purdue University, United States of America

## Abstract

The emerald ash borer (*Agrilus planipennis*) is an invasive wood-boring beetle that has killed millions of ash trees since its accidental introduction to North America. All North American ash species (*Fraxinus* spp.) that emerald ash borer has encountered so far are susceptible, while an Asian species, Manchurian ash (*F. mandshurica*), which shares an evolutionary history with emerald ash borer, is resistant. Phylogenetic evidence places North American black ash (*F. nigra*) and Manchurian ash in the same clade and section, yet black ash is highly susceptible to the emerald ash borer. This contrast provides an opportunity to compare the genetic traits of the two species and identify those with a potential role in defense/resistance. We used Difference Gel Electrophoresis (DIGE) to compare the phloem proteomes of resistant Manchurian to susceptible black, green, and white ash. Differentially expressed proteins associated with the resistant Manchurian ash when compared to the susceptible ash species were identified using nano-LC-MS/MS and putative identities assigned. Proteomic differences were strongly associated with the phylogenetic relationships among the four species. Proteins identified in Manchurian ash potentially associated with its resistance to emerald ash borer include a PR-10 protein, an aspartic protease, a phenylcoumaran benzylic ether reductase (PCBER), and a thylakoid-bound ascorbate peroxidase. Discovery of resistance-related proteins in Asian species will inform approaches in which resistance genes can be introgressed into North American ash species. The generation of resistant North American ash genotypes can be used in forest ecosystem restoration and urban plantings following the wake of the emerald ash borer invasion.

## Introduction

The emerald ash borer (EAB), *Agrilus planipennis* Fairmaire (Coleoptera: Buprestidae), is an invasive insect that has killed tens of millions of ash (*Fraxinus* spp.) trees in the U.S. and Canada [1], [2]. Larvae feed on phloem and outer xylem of host trees, which disrupts translocation of water and nutrients and results initially in canopy thinning and ultimately death within one to three years of first expression of symptoms [2], [3], [4], [5]. All North American species of ash that emerald ash borer has encountered thus far are susceptible to colonization, even when growing on high quality sites and in the absence of obvious environmental stress [3], [6], [7].

Conversely, Asian species of ash, which share a coevolutionary history with emerald ash borer, appear to be colonized only when weakened by abiotic or biotic stress [8], [9]. In a common garden experiment, Rebek et al. [6] found Manchurian ash (*F. mandshurica* Ruprecht), which is a primary host in its endemic range, to be much more resistant to emerald ash borer than North American green (*F. pennsylvanica* Marsh) and white ash (*F. americana* L.). A Manchurian x black ash (*F. nigra* Marsh) cross was also highly susceptible, indicating the hybrid did not inherit emerald ash borer resistance from its Asian parent. North American black ash is also known to be highly susceptible. In Michigan forests, for example, mortality of black ash proceeds at a faster rate than green and white ash [10].

Resistance of deciduous trees to wood-boring insects is hypothesized to be the result of a combination of constitutive and induced, physical and phytochemical defenses that deter or kill the insect [11]. Constitutive defense traits that confer resistance to wood-borers, such as defensive phytochemicals or proteins, could serve as biomarkers for use in introgressing emerald ash borer resistance genes into North American ash species via hybridization or transgenic approaches. Previous work identified differences in the constitutive phenolic chemistry of phloem tissues for Manchurian, green, and white ash [12], [13]. However, information about putative resistance-related defensive proteins is lacking.

While insightful information can be obtained at the level of gene sequence and gene expression (genomics and transcriptomics, respectively), it is ultimately the proteome and the products of enzymatic reactions that dictate the interaction between plant and herbivore. Proteins that mediate plant-insect interactions include those that confer resistance directly (e.g. cysteine proteases or proteinase inhibitors) or indirectly through their roles in defense pathways (e.g., enzymes involved in the biosynthesis of defensive phytochemicals) [14]. Therefore, proteins serve as a logical starting point in the search for putative resistance genes.

One approach to investigate putative constitutive resistance traits is to use high-throughput methods to compare susceptible and resistant hosts. Proteomic high-throughput methods include techniques, such as difference gel electrophoresis or DIGE, that provide qualitative and quantitative information on total proteomic differences between two or more experimental units [15]. Information garnered from DIGE studies can serve as the basis for functional experiments in which resistance genes can be characterized *in planta* using transgenic approaches or through the use of Asian x North American ash hybrids coupled with in depth analyses of the interaction between the modified/hybrid plant and the pest.

**Figure 1:**
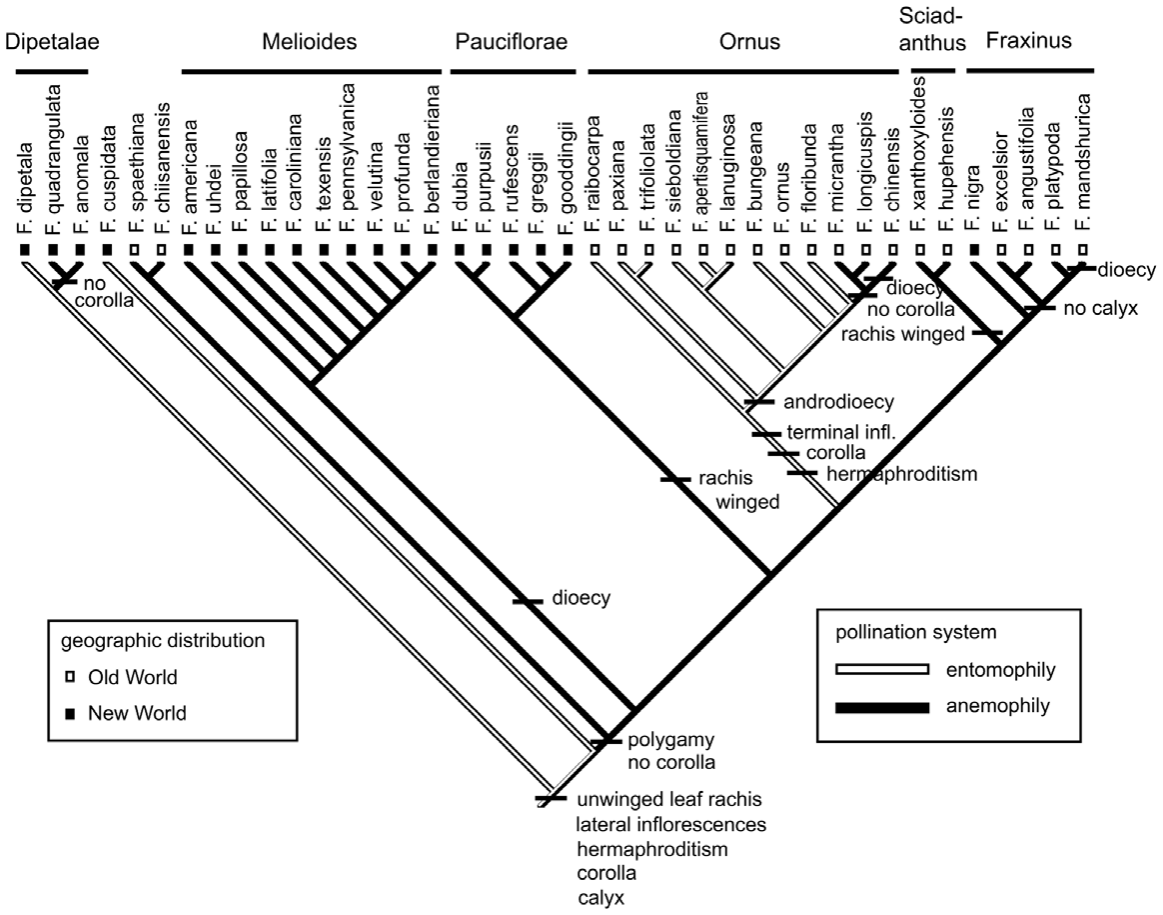
Phylogenetic tree of the genus *Fraxinus*. Species used in this study are outlined. Reproduced with permission from Eva Wallander and Springer publishing.

In this study, the constitutive proteome of whole phloem tissue of Manchurian ash was compared to that of three susceptible North American ash species. A recent phylogenetic analysis of the genus *Fraxinus*, based on DNA sequences from the nuclear ribosomal ITS and two chloroplast regions of all 43 species, placed black and Manchurian ash in the same clade in the section Fraxinus (Figure 1) [16]. The contrast between phylogenetic similarity and divergence in phenotype of resistant Manchurian and susceptible black ash to emerald ash borer provides an opportunity to investigate their genetic differences in order to identify potential resistance traits.

DIGE has not been used extensively to study interspecific variation [17]. To identify biologically meaningful proteins in Manchurian ash phloem tissues, we first compared its proteome to that of black ash. Further filtering against the more phylogenetic dissimilar, yet susceptible, green and white ash was conducted to strengthen our selection of resistance-related gene candidates (Figure 1).

## Materials and Methods

### Experimental Design

Clonal individuals of *F. mandshurica* cv. ‘Mancana’, *F. nigra* cv. ‘Fallgold’, *F. pennsylvanica* cv. ‘Patmore’, and *F. americana* cv. ‘Autumn Purple’, were obtained from Bailey Nursery, Inc., St. Paul MN, USA. Six-yr-old saplings of each species were planted in a common garden established in November 2007 in Bowling Green, OH. Trees were planted in a randomized complete block design with eight blocks. We sampled one sapling per block of each species for a total of 8 biological-clonal replications per species were sampled on August 6, 2008. At sampling, Manchurian, black, green, and white ash trees had mean stem diameters of 3.4±0.06 (S.E.M.) cm, 2.9±0.1 cm, 3.6±0.04 cm, and 3.3±0.1 cm respectively, at 30 cm above the soil line. Second year branches were chosen for analysis. Branches were removed from trees, stripped of leaves, placed on ice, and then transported back to the lab where phloem tissue was immediately removed, frozen in liquid nitrogen, and stored at −80°C until protein extraction.

### Protein Extraction and Purification

Proteins were extracted according to Vâlcu and Schlink [18], with minor modifications to accommodate for differences in scale, protein extract preparation, and cleaning for DIGE experiments. Phloem tissue was ground in liquid nitrogen and 0.1 g was suspended in 500 µl of pre-cooled (−20°C) precipitation solution: 10% TCA (Sigma-Aldrich; St. Louis, MO, USA) and 20 mM DTT (BioRad; Hercules, CA, USA) in acetone. Proteins were precipitated overnight at −20°C. Phloem tissue and precipitated proteins were then washed twice for 1 h each at −20°C with 1 ml of 20 mM DTT in acetone and pelleted by centrifugation for 30 min at 26 000×g (4°C). Following removal of supernatant, the pellet was dried under vacuum for 10 min and re-extracted twice with 500 and 200 µl of extraction buffer [7 M urea (BioRad), 2 M thiourea (Sigma-Aldrich), 4% CHAPS (BioRad), 50 mM DTT (BioRad), and 1x Complete Protease Inhibitor cocktail (Roche; Indianapolis, IN, USA)]. Protein extracts were then subjected to two subsequent purification steps prior to DIGE in order to remove remaining contaminants (i.e. salts, sugars, and secondary plant compounds). For the first clean-up step, proteins were precipitated from the protein extracts using the Bio-Rad ReadyPrep™ 2-D Clean-up Kit and re-suspended in 25 µl of a DIGE compatible buffer [7 M urea, 2 M thiourea, 4% CHAPS, 30 mM Tris (pH 8.5), and 1x Complete Mini EDTA-free Protease Inhibitor Cocktail (Roche)]. Protein pellets were placed on a shaker at RT for 3 h to allow for complete re-solubilization of the pellet. Protein concentrations were measured with the Coomassie Plus™ Protein Assay (Pierce; Rockford, IL, USA) and compared against a standard curve of BSA prepared in a DIGE compatible buffer. We ran a 1-D protein gel to ensure that the integrity of the proteins was not affected (Figure S1).

### 1-D SDS-PAGE

A 1-D protein gel was run to ensure that the integrity of the proteins was not affected (Figure S1). Twenty µg of ash phloem protein samples were separated under denaturing conditions in a discontinuous sodium dodecyl sulfate polyacrylamide gel (SDS-PAGE) (stacking gel – 3.5%; resolving gel – 12%). Before separation on the gel, ash protein samples were diluted in Laemmli buffer (1∶1). The SDS-PAGE gel was run in a Mini Protein 3 Cell (Bio-Rad, CA, USA) at 75 V for 30 min and 150 V for 1 h. After completion of electrophoresis protein bands were visualized in the gel via Coomassie blue staining.

### Sample preparation for DIGE

Prior to running DIGE, a second protein clean-up step was necessary to remove residual contaminants that were observed on a 2-D SDS-PAGE gel used to visualize proteins prior to the DIGE experiment (data not shown). Protein extracts were precipitated out of solution with a 4∶1 (v∶v) methanol: chloroform mixture in order to remove residual sugars. Following precipitation, protein pellets were re-suspended in lysis buffer [7 M urea, 2 M thiourea, 4% CHAPS, 30 mM Tris (pH 8.5)]. Proteins were then quantified via the Bradford assay using BSA as the standard and diluted to a concentration 1 µg/µl. We randomly labeled each of the eight replicate protein extracts for each species with 8 pM of either Cy3- or Cy5- minimal labeling dyes (GE Healthcare; Little Chalfont, UK) in DMF (N, N – dimethylformamide) for every 1 µg of protein. Following calibration, the internal standard (IS), consisting of 22 µl from each sample, was pooled into one tube (total 704 µl) and labeled with Cy2- minimal labeling dye in the same proportions as with the other samples. Labeled samples were allowed to react on ice for 30 min. After 30 min, 1 µl of 10 mM lysine was added to each sample to quench the labeling reaction. Labeled samples (a total of 40 µg from each sample) were then combined as shown in Table S1, vortexed, and diluted with sample buffer [7 M urea, 2 M thiourea, 2% CHAPS, 65 mM DTT and 0.5% immobilized pH gradient (IPG) buffer (GE)] to a volume of 470 µl before loading on the IPG strip.

### IPG Strip p*I* Range and First Dimension Isoelectric Focusing

**Figure 2:**
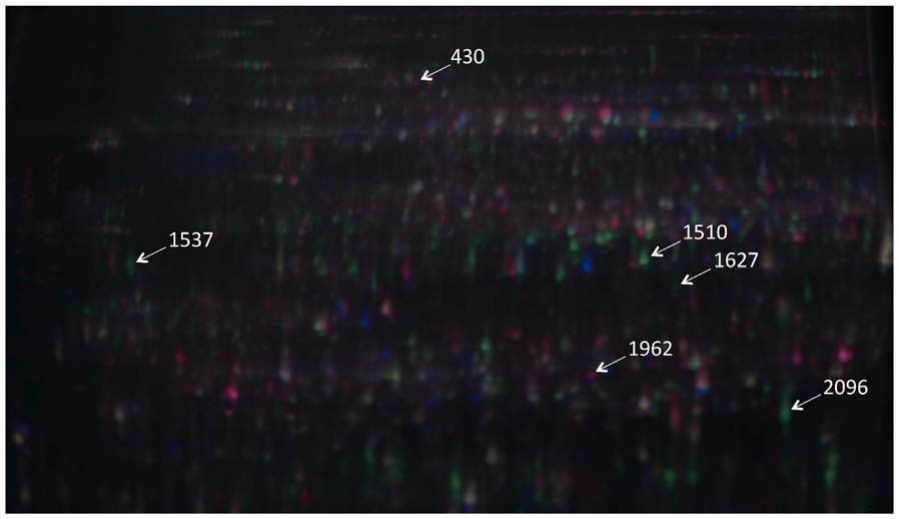
2-D DIGE master gel overlay. Gel image (*pI* 4–7) showing proteins derived from Manchurian ash (Cy3 – red spots) and black ash (Cy5 – green spots). The internal standard (composed of equal parts from all ash protein extracts) is displayed as blue spots. Yellow spots are those common to all species. Numbered spots identify putative defense and susceptibility related proteins (see Tables 2 and 3).

We used a 2-D SDS-PAGE gel using an IPG strip with a p*I* range of 3–10 was used to determine the optimum range of proteins in our extracts (Figure S2). The majority of proteins were found to be concentrated in the 4–7 p*I* range. IPG strips (p*I* 4–7) were then used for all subsequent analyses in order to achieve better protein spot resolution (Figure 2). Prior to isoelectric focusing, samples were applied to 24 cm IPG strips (p*I* 4–7), overlaid with mineral oil, and allowed to rehydrate passively overnight. Isoelectric focusing (IEF) was performed using an Ettan™ IPGphor™ II Isoelectric Focusing System (GE Healthcare/Amersham Biosciences). Isoelectric focusing took place using the following conditions: instrument temperature set at 20°C; maximum current set at 75 µA/strip; step (1): step and hold at 500 V for 1 hr (500 total Vhr); step (2): rapid ramping up to 1000 V for ∼1 hr (800 total Vhr); step (3): rapid ramping up to 10,000 V for 3 hr (16,500 total Vhr); step (4): hold at 10,000 V up to a total of 52,500 Vhr for 3.5 hr. After focusing, strips were stored at −80°C until used in the second dimension analysis.

### Strip Equilibration and Second Dimension

Prior to running the second dimension gels, IPG strips were thawed, reduced in equilibration buffer I (6 M urea, 30% glycerol, 75 mM Tris pH 8.8, 2% SDS, and 0.5% DTT), and alkylated in equilibration buffer II (6 M urea, 30% glycerol, 75 mM Tris pH 8.8, 2% SDS, and 4.5% iodoacetamide) for 15 min in each. Following equilibration, we rinsed IPG strips in SDS running buffer (25 mM Tris, 192 mM glycine, 0.2% SDS), and sealed on top of 12% SDS-PAGE gels (26×20×0.1 cm). Gels were then overlaid with 0.5% w∶v agarose and a trace amount of bromophenol blue in running buffer, and run at a constant 2 W at 20°C for 45 min and constant 15 W per gel for 4.5 hr in SDS running buffer (25 mM Tris, 192 mM Glycine, 0.1% SDS).

### Gel Imaging and Statistical Analysis

**Figure 3:**
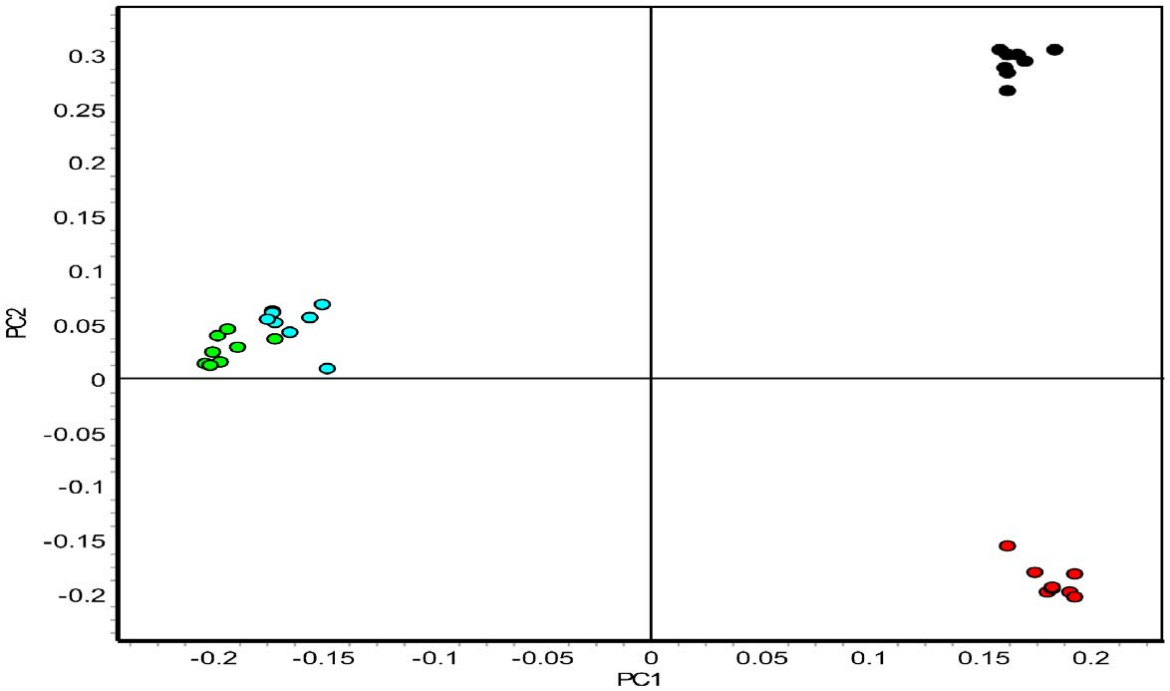
PCA analysis of ash phloem proteomic profiles. PC1 clearly separates black (black circles) and Manchurian (red circles) ash from white (blue circles) and green (green circles) ash, while PC2 separates Manchurian and black ash but not white and green ash. Each color point represents a single biological replicate (n = 8/species).

Following SDS-PAGE, gels were scanned using a Typhoon Variable Mode Imager 9400 (GE Healthcare). Cy2-, Cy3-, and Cy5- labeled protein images were produced by excitation of gels at 488, 532, and 633 nm, respectively and emission at 520, 590, and 680 nm, respectively. Gel images were analyzed statistically using DeCyder v6.05.11 software (GE Healthcare). A total of eight biological replicates/species were used for all statistical analyses. Each image (32 analytical images and 16 IS images) was manually analyzed to exclude saturated spots, artifacts (dust spots), and noise. We chose the IS image of gel 10 as the master gel because of the gel quality and total number of spots detected (2,434). The settings used to detect spots were optimized using the master gel as a reference and a number was assigned to each spot. The same settings were then used for all other gel image analyses. Boundary and volume of protein spots were detected according to the Decyder spot detection algorithm. Statistical analyses were performed as follows: normalized protein volume ratios were calculated (DIA; differential in-gel analysis module) for each individual protein spot from Cy3- and Cy5- sample relative to Cy2- (IS) corresponding to the same spot. These values were used for further statistical analyses and referred to as the standardized abundance. Differences in average standardized abundances between experimental groups show differential protein expression and are expressed as an average ratio. One-way ANOVA was performed using the Decyder software (which also simultaneously controls for false discovery rate (FDR)) to evaluate differences in protein expression between all 4 experimental groups. We used the same analysis to evaluate differences between only two experimental groups (i.e. black vs. Manchurian, green vs. Manchurian, and white vs. Manchurian) as an equal variance, two-tailed Student's t-test. Proteins of interest (POI) were defined as having abundances that were significantly different (*P*<0.05) between: 1) Manchurian and black ash, 2) Manchurian and green ash, and 3) Manchurian and white ash experimental groups along with an absolute abundance ratio that was greater than 2-fold. We chose to focus on the 355 POI from Manchurian and black ash meeting these criteria for sequencing and identification as black and Manchurian ash share the most recent common ancestor and are therefore closely related phylogenetically (Figure 1 and Table 1) [16], [19]. We further limited our selection by comparing the average ratios of green and white ash in order to remove proteins that did not differ significantly (*P*<0.05; 2-fold or greater) when compared to proteins identified from the Manchurian and black ash comparison. Principal component analysis (PCA) was used to separate experimental groups and was performed using the algorithm provided with the Decyder software (Figure 3).

**Table 1:** Number of proteins differentially expressed between North American ashes and Manchurian ash.

Comparison	Number of Proteins Significantly Different^a^
White vs. green	215
Manchurian vs. black	355
White vs. black	545
White vs. Manchurian	580
Green vs. black	589
Green vs. Manchurian	610

Differences between species reflect phylogenetic relatedness (Figure 1).

a >2-fold absolute difference and *P*<0.01.

### Preparative Gel, Protein Spot-cutting, and Trypsin Digestion

Equal amounts of unlabeled individual biological replicates were pooled for each species and loaded on preparative gels for a total of 400 µg/gel. We ran two preparative gels using the above conditions for first and second dimensions. Preparative gels were fixed (15% ethanol, 1% citric acid) overnight and stained with LavaPurple (FLUOROtechnics). Gels were de-stained using 15% ethanol for 1 hr. LavaPurple stained gels were scanned using a Typhoon Variable Mode Imager 9400 (GE Healthcare) with 532 nm excitation wavelength and 610 nm emission filter and matched to the master gel image. We subjected the 355 POI to further analysis, of which 264 were successfully matched to the master gel image and used to generate a picking list. Proteins of interest were excised from the gels using the Ettan Spot Picker in conjunction with the Ettan Spot Handling Workstation (GE Healthcare). Spots were digested with trypsin using the Ettan Digester robot (GE Healthcare) in preparation for peptide sequencing using Nano-LC-MS/MS.

### Nano LC-MS/MS, Protein Identification, and Data Deposition

Tryptic peptides were sequenced *via* capillary nano-LC-MS/MS on a Thermo Finnigan LTQ mass spectrometer equipped with a nanospray source operated in positive ion mode. Capillary nano-LC-MS/MS was performed using similar methods as described in [20]. Solvent A (50 mM acetic acid in water) and solvent B (acetonitrile) were used for all chromatographic separations. Samples (5 µl from each sample) were prepared in solvent A and injected onto a µ-Precolumn Cartridge (Dionex, Sunnyvale, CA) and washed with 50 mM acetic acid. The injector port was switched to inject and the peptides were eluted from the trap onto the column. A 5 cm, 75 µm ID ProteoPep II C18 column (New Objective, Inc. Woburn, MA) packed directly in the nanospray tip was used for all chromatographic separations. Peptides were eluted directly off the column into the LTQ system using a gradient of 2–80% solvent B over 45 minutes, with a flow rate of 300 nl/min. The total run time was 65 minutes. We operated the nanospray source with a spray voltage of 3 kV and used a capillary temperature of 200° C was used. The scan sequence of the mass spectrometer was based on the TopTen™ method; the analysis was programmed for full scan (recorded between 350–2,000 Da), and a MS/MS scan to generate product ion spectra to determine amino acid sequence in consecutive instrument scans of the ten most abundant peaks in the spectrum. The CID fragmentation energy was set at 35%. We enabled dynamic exclusion with a repeat count of 2 within 10 seconds, a mass list size of 200, an exclusion duration of 350 seconds, a low mass width of 0.5, and a high mass width of 1.5. We converted the raw data files collected using the mass spectrometer to mzXML and MGF files using MassMatrix data conversion tools version 1.3 (http://www.massmatrix.net/download). For low mass accuracy data, tandem MS spectra that were not derived from singly charged precursor ions were considered as both doubly and triply charged precursors. The resulting MGF files were searched using Mascot Daemon by Matrix Science version 2.2.2 (Boston, MA). The .mgf files were searched against the NCBI database version 20090710 limited to Viridaeplantae (green plants) as taxonomy (384,871 sequences). Trypsin was selected as the digest enzyme with up to two missed cleavages. Carbamidomethyl and oxidation were set as the fixed and variable modifications. Peptide and fragment mass tolerances were set to ±2 Da and 0.8 Da respectively. Sequence data were also automatically searched against a decoy database in order to avoid false positives. MASCOT based probability scores were used to evaluate protein identities and were considered correct if the match had a score greater than 70, which indicates identity or significant (*P*<0.01) similarity, and two peptide matches. Identities were accepted in some cases if the above parameters were met and only one peptide match was found along with an expected value that was highly significant (*P*<0.001) for the peptide match and the MASCOT protein score was 70 or greater for that single peptide match. We checked all protein identities reported in this paper manually to confirm –b and –y ion sequence tags in MS/MS spectra. Any protein spot to match two or more protein identities in the MASCOT database search (meaning more than one protein was potentially present in the cored spot), or did not pass any of the above criteria, was not used in further analyses. Filtered peptide data have been deposited with Peptidome, National Center for Biotechnology Information. All information and raw data associated with peptides identified in Manchurian, black, green, and white ash are accessible on the Peptidome NCBI Peptide Data Resource homepage (http://www.ncbi.nlm.nih.gov/peptidome) via study accession number PSE148. Specific information about individual peptides identified from black, green, white, and Manchurian ash are accessible directly through sample accession numbers PSM1313 and PSM1314.

### Protein Gene Ontology Annotation and Identification of Putative Defense and Increased Susceptibility Genes

Gene Ontology (GO) annotations for biological processes were added to all proteins with an average fold-change of 2 and above (*P*<0.05) across all species comparisons (Tables S2 and S3). Gene ontologies were added by searching individual gi numbers (obtained from the MASCOT search files) in the Protein Information Resource Database (National Biomedical Research Foundation, pir.georgetown.edu, Washington, D.C.). Proteins that were not found in the PIR database were subsequently searched in the NCBI databank to obtain a basic biological understanding of the protein. Most proteins were associated with multiple biological process GO terms and those not associated with a biological process GO term had a molecular function term that was recorded. Proteins that did not have a biological process GO term and did have a molecular function GO term was treated as miscellaneous proteins in this paper (Table S2 and Table S3). We selected a single biological process category for proteins associated with multiple GO terms by choosing the most specific and biologically relevant term to plant defense against herbivores. We loaded specific biological process GO categories for black, green, white, and Manchurian ash onto the QuickGO [21] annotation page in order to visualize relationships between biological processes for each species independently. To organize the proteins identified in this study and visualize the ontology distribution of proteins significantly differing (*P*<0.05) with an absolute abundance ratio greater than 2-fold between Manchurian, black, green, and white ash we used a limited subset of high-level GO terms as parent categories (Table S2 and Table S3).

Potential resistance-related genes in Manchurian ash were selected by using similar criteria as described in [17]. Potential constitutive resistance-related proteins were selected based on the following criteria: 1) an absolute abundance ratio greater than 5-fold when expression in Manchurian ash was compared to expression across all three susceptible species of ash, 2) a highly significant difference (*P*<0.05) in expression was found when compared to the three susceptible ash species, and 3) the protein's potential direct or indirect role in plant resistance based on its gene ontology annotation as it relates to the known literature of plant defense. Conversely, proteins potentially related to enhanced susceptibility to EAB were chosen based on: 1) an absolute abundance ratio greater than 5-fold when expression in black, green, and white ash was compared to Manchurian ash protein expression, and 2) a highly significant difference (*P*<0.05) in expression was found when all three susceptible ash species were compared to Manchurian ash.

## Results

A total of 2,434 spots were detected in the master gel (Figure 2). The internal standard image of gel 10 (Figure 2 and Table S1) was chosen as the master gel image that consisted of equal parts of protein extracts from each individual biological replicate from all four species of ash. An average of 2,184 (±71 spots [95% confidence interval]) spots were detected in all 16 gels. We were able to resolve 1,733 (±100 spots [95% confidence interval]) of the 2,434 spots detected in the master gel (*P*<0.01) for all 16 gels, each containing two separate biological replicates plus the internal standard (Figure 2). The 1,733 spots that were matched to the master gel image on each gel were used for all subsequent statistical analyses.

### Principal Component Analysis

Principal component analysis (PCA) revealed a clear separation between species in terms of proteins found to differ (*P*<0.05) in abundance, and with an absolute abundance ratio greater than 2-fold (n = 8 for each species) (Figure 3). White and green ash co-localized in the same region of the PCA grid while individual replicates grouped together by species. Black and Manchurian ash co-localized in the same plane (PC1) but were separated by PC2. PC1 and PC2 combined to account for nearly 64% of the original variance, with 46.5% explained by PC1 and 17.3% explained by PC2. Results of the PCA suggest that DIGE is a robust methodology to make interspecific proteomic comparisons.

### Proteomic Differences between Manchurian and Black, Green, and White Ash

A total of 355 proteins were found to differ (*P*<0.01) between Manchurian and black ash with an absolute abundance ratio greater than 2-fold (Table 1). Of these, 178 proteins had a higher level of differential expression in black ash (131 proteins ranging between 2–5-fold and 47 proteins with a > 5-fold difference), while 177 had higher levels of expression in Manchurian ash (126 proteins ranging between 2–5-fold and 51 proteins with a > 5-fold difference). A total of 264 of the initial 355 proteins of interest could be reliably matched from the preparative gels to the master gel image and were included in the picking list generated for nano-LC-MS/MS analysis. Based on the following criteria, we excluded 147 of the 264 identified proteins of interest from the comparison between black and Manchurian ash based on: 1) multiple protein identities were detected for a single spot, 2) protein identities assigned to a protein spot did not meet our criteria for a reliable match based on MASCOT and statistical data, or 3) no protein identity was assigned for the spot in question due to the lack of a sufficient match in the MASCOT database search. We also incorporated data related to the comparison of Manchurian vs. green, Manchurian vs. white, green vs. Manchurian, and white vs. Manchurian ash species to further eliminate an additional 65 proteins based on no significant difference of differential expression when compared to these species. Using the comparisons of Manchurian against green and white we identified 33 proteins that had a significantly higher (*P*<0.05; > 2-fold) expression when compared across all species (Table S2). We used similar criteria to identify 19 proteins present in the 3 susceptible species with a > 2 –fold level of differential expression (*P*<0.05) when compared to Manchurian ash (Table S3). These eliminations resulted in a total of 52 proteins from the initial 355 proteins of interest [33 significantly (*P*<0.01; > 2-fold average ratio) more abundant in Manchurian ash and 19 significantly more abundant in black, green, and white ash] were included in the final analysis (Table S2 and Table S3).

### Protein Identification, Classification, and Gene Ontology Annotation

Proteins were classified according to gene ontology (GO) annotations derived from graphical representations of biological process information using GO Slim (Table S2 and Table S3). A limited subset of high-level GO terms (9 total categories and a miscellaneous category based on information obtained from the QuickGO resource) was used to organize the proteins identified in this study as they related to one another based on their GO annotation relationships (Table S2 and Table S3). Manchurian ash proteins 502 and 1481 had no associated biological process GO term, but were associated with hydrolase and lactoylglutathione lyase activity for molecular function GO terms, respectively (Table S2). Black/green/white ash had two proteins (1032 and 1722) with no associated GO term for any category. The remaining miscellaneous protein (975) was associated with ATP binding activities (Table S3).

### Identification of Putative Defense and Susceptibility-Related Genes

**Figure 4:**
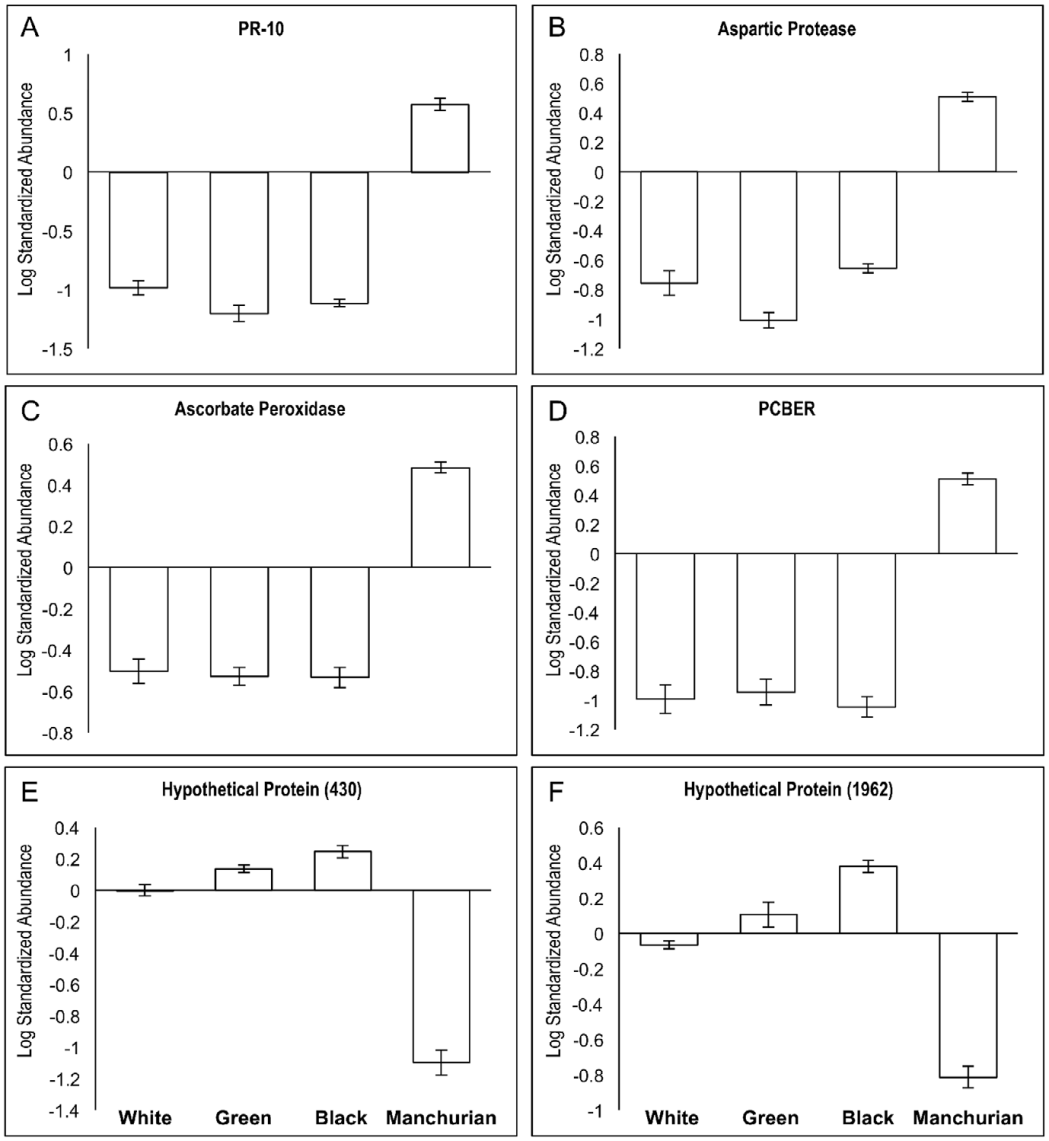
Bar graphs of mean log standardized abundance (LSA) values for the proteins shown in **Tables 2** and **3**. Panels A, B, C, D: Proteins expressed at higher levels (> 5-fold; *P*<0.01) in resistant Manchurian (M) ash than in susceptible North American black (B), white (W), and green (G) ash. Panels E and F: Proteins expressed at higher levels (> 5-fold; *P*<0.01) in susceptible black, green, and white ash than resistant Manchurian ash. Bars represent the mean of eight biological replicates within a genotype/species, while error bars represent the standard error of the mean (s.e.m.). The zero value for LSA corresponds to the internal standard (IS). N = 8, except in Manchurian ash PR-10, where n = 7 due to a lack of a match for this protein in gel 1 (Table S1) to the master gel image.

The criteria used to compare ash species were similar to those of a previous DIGE study that compared the proteomes of two *Entamoeba* species [17]. An absolute abundance ratio cutoff of 5-fold (with *P*<0.05) between Manchurian and black, green, and white ash resulted in the selection of a total of 13 proteins present in Manchurian ash phloem as putative constitutive resistance-related proteins. Information relating to GO annotations for biological process were used to identify proteins with a putative role in plant defense, based on the current literature and reduced the selection to four proteins (1510, 1537, 1627, and 2096) (Table 2 and Table S2). Conversely, two proteins (430 and 1962) fit these criteria for the green, white, and black ash/Manchurian ash comparison (Table 3 and Table S3) and are discussed below. The four proteins we identified (PR-10, aspartic protease, phenylcoumaran benzylic ether reductase, and ascorbate peroxidase) with potential for a direct or indirect role in resistance to emerald ash borer are discussed below (Table 2). There was very little variation in protein expression among individual biological replicates of Manchurian, black, green, and white ash for these four proteins with Manchurian ash having significantly higher quantities than when compared to the three susceptible ash species (*P*<0.001) (Figure 4).

**Table 2:** Phloem proteins putatively related to defense identified in Manchurian ash.

Protein Master Gel Number	Average Ratio (*P*-value)*			MASCOT Score [No. of peptides]	NCBI Accession Number‡	Protein ID	Annotation†
	M/B	M/G	M/W				
2096	49.85(7.10E^−11^)	56.74 (2.1E^−10^)	35.31 (3.0E^−9^)	76 [1]	gi|886683	Major Allergen [*Malus x domestica*]	Defense Response
1510	33.57 (1.6E^−9^)	25.6 (1.4E^−9^)	27.44 (6.0E^−9^)	435 [9]	gi|7578895	Phenylcoumaran benzylic ether reductase Fi1 [*Forsythia x intermedia*]	Metabolic Process
1537	14.52 (6.50E^−11^)	31.42 (1.3E^−11^)	16.28 (6.3E^−9^)	78 [2]	gi|13897888	Putative Aspartic Protease [*Ipomoea batatas*]	Proteolysis
1627	10.15 (2.60E^−09^)	10.03 (6.5E^−11^)	9.18 (2.5E^−9^)	110 [2]	gi|25992557	Thylakoid-Bound Ascorbate Peroxidase [*Triticum aestivum*]	Hydrogen Peroxide Catabolic Process

*Proteins present in higher abundances (> 5-fold) in Manchurian ash relative to black, green, and white ash.

† Annotations are Gene Ontology annotations for Biological Process.

‡ Searching NCBI Peptidome using the NCBI accession number of the matched protein will lead to detailed information about the peptides identified in this study. Manchurian ash peptide information can be obtained through Peptidome sample accession number PSM1314.

## Discussion

In this study we describe the first analysis of phloem tissues from a tree species using an interspecific comparative proteomics approach for the purpose of identifying potential constitutive resistance/susceptibility-related genes against an invasive insect pest. Overall, interspecific variation in the phloem proteome corresponded strongly to phylogenetic relationships between species (Figure 1, Figure 3, and Table 1). Using this approach, we identified four proteins that may play a role in the resistance of Manchurian ash to emerald ash borer: a major allergen (PR-10), a putative aspartic protease, a phenylcoumaran benzylic ether reductase, and a thylakoid-bound ascorbate peroxidase. Using the same criteria to select proteins of interest from black ash, we identified two putative susceptibility-related genes.

### Proteomic Analysis of Non-Model Plants and Inter-species Comparisons Using DIGE

Protein sequences are highly conserved across organisms. This offers a major advantage for the high-throughput identification of gene products of non-model plant species via comparison to well known protein orthologs [22]. Furthermore, monitoring changes in global gene expression is emerging as an important tool for dissecting the molecular basis of plant interactions with other organisms [23]. Therefore, studying proteins on a global scale using a proteomic approach can give insight into specific biological processes taking place in an organism or tissue at any one point in time [24]. Proteomic analysis of non-model plants is necessary to understand specific features and processes that are unique to certain plant systems which cannot be answered by model organisms [24]. Proteomic analysis has been applied successfully to several woody plant species in order to understand fundamental processes about wood formation, mechanisms governing fruit ripening, and responses to abiotic stress [25], [26], [27]. However, only a few studies have used proteomic methods (conventional 2-DE or DIGE) to compare different species [17], [28]. As others have noted, the challenges associated with cross-species analysis using a two-dimensional approach are not trivial. Amino acid sequence substitutions resulting from genomic differences between two closely related species, as well as post-translational modifications, splice variants, insertions, etc. could affect the location of a protein on a two dimensional plane via differences in their molecular weight and isoelectric point [17].

Further challenges in comparing the proteomes of ash species emerge from their lack of genomic information. Nonetheless, we did find that the total proteomic differences between ash species (Table 1) bear a strong correspondence with phylogenetic relationships (Figure 1) [16]. Furthermore, PCA revealed a clear separation between species that also corresponded with their evolutionary relationships (Figure 3). In order to detect differences between species, a more conservative approach to visualizing differential expression is required. For instance, we focused on proteins that showed at least a 2-fold difference in expression between species for PCA (Figure 3) and total proteomic analysis (Table 1), as compared to the 1.5-fold difference criterion employed in a typical DIGE study [27]. Clearly, DIGE efficiently detected proteomic differences between species. However, ascribing functional roles to individual proteins is more difficult. Proteins known to contribute to virulence in humans were identified by comparing the constitutive proteomic differences between a virulent and a closely related non-virulent protozoan species using DIGE [17]. The identification of these proteins was accomplished consistently when the authors focused only on proteins with much higher levels of differential expression (≥5-fold difference, *P*<0.01) in the pathogenic species when compared to the non-pathogenic relative. By using the same criteria, we were able to identify four unique genes from Manchurian ash with a potential role in defense against the emerald ash borer and two in susceptible ash species that may contribute to their susceptibility.

### Putative Resistance-Related Genes in Manchurian Ash

#### Major Allergen

The major allergen (PR-10) had the highest average ratio for any protein found in Manchurian ash when compared to the susceptible ash species. The major allergen from apple (*Malus domestica* L. Borkh.), Mal d 1, which best matched the protein from Manchurian ash, is related to the birch family of allergens (Bet v 1). Mal d 1 is a pathogenesis-related (PR) protein, which suggests a potential role in plant defense against microbial attack and stress tolerance [29], [30]. While classification of proteins as PR requires induction in response to microbial attack or related phenomena, many PR proteins are expressed constitutively in plant tissues, i.e. with no association to biotic attack [30], [31]. While PR-10 proteins are induced in response to a wide range of plant pathogens [32], [33], [34], their role in host resistance to insects has not been studied. However, PR-10 protein expression appears to be regulated by methyl jasmonate (MeJA) in several plant species [35], [36]. MeJA, and ultimately jasmonic acid-isoleucine conjugates (JA-Ile), regulate an important signaling pathway in the elicitation of induced resistance to herbivorous insects [14]. In Manchurian ash, MeJA also has been shown to mediate emission of volatile compounds, suggesting the MeJA pathway is active and potentially regulates defense responses in this species [37]. PR-10 proteins have a diverse array of biological functions that include antimicrobial [38], ribonuclease [39], ligand-binding activities [40], and involvement in secondary metabolism [41]. The very high differential expression of constitutive PR-10 in Manchurian ash phloem relative to black ash suggests a potential role, either direct and/or indirect, in resistance of Manchurian ash to emerald ash borer.

#### Phenylcoumaran Benzylic Ether Reductase

Phenylcoumaran benzylic ether reductases (PCBER) are enzymes involved in neo-lignan biosynthesis [42]. Lignoids (lignans and neo-lignans) are a class of phenolic metabolites found throughout the plant kingdom with documented roles in plant defense [43], [44]. In relation to insects, lignans are known to have feeding and growth inhibition activities as well as toxicity against insects [44], [45], [46]. The presence of lignans in *Fraxinus* spp. has been documented extensively [12], [47], [48]. More specifically, lignans were found to be much more highly concentrated in the phloem tissues of Manchurian ash when compared to green and white ash [12], [13]. PCBER accumulates in the cambial region of young stems of *Forsythia intermedia* (a member of the Oleaceae, and relative to the genus *Fraxinus*) and has been implicated as serving dual functions as synthesizing key components for plant growth and active defense [49], [50]. PCBER also acts directly as a food allergen and as a result is classified as being related to pathogenesis-related proteins [51]. Based on the high level of expression of PCBER in Manchurian ash phloem and the current literature regarding the direct and indirect functions in plant defense; PCBER is a very good candidate for future functional characterization as it relates to resistance against emerald ash borer.

#### Aspartic Protease

The putative aspartic protease we identified was consistently more highly expressed in Manchurian ash phloem than in the North American species of ash. Aspartic proteases have been found in all kingdoms of life, but our understanding of their biological roles derives mostly from microbes and animals [52], [53]. In plants, only serine proteases are more abundant than aspartic proteases [54]. The latter have been found in monocots, dicots, and gymnosperms [55], [56], [57] where they are typically associated with distinct organs, depending on species [58], [59]. Aspartic proteases in plants are involved in protein processing and degradation, senescence, stress responses, programmed cell death, reproduction, and antimicrobial defenses [52], [53], [54], [60], [61], [62]. In potato (*Solanum tuberosum* L.) tubers and leaves, aspartic proteases display dose-dependent antimicrobial activity and are induced in response to infection by *Phytophthora infestans* and mechanical wounding [56], [59], [62], [63]. In corn, cysteine protease has been shown to confer resistance to fall armyworm (*Spodoptera frugiperda*) *via* degradation of the peritrophic membrane of this chewing insect, which interferes with nutrient acquisition, ultimately killing the insect [64], [65], [66]. Aspartic proteases are therefore extremely diverse in their roles in other biological systems and may participate in ash defense against emerald ash borer with mechanisms similar to those played by cysteine proteases in corn, or others yet to be characterized.

#### Thylakoid-Bound Ascorbate Peroxidase

Resistance of Manchurian ash to the emerald ash borer may also be mediated by ascorbate peroxidase. In plants, ascorbate peroxidases scavenge radical oxygen species during photosynthesis. Herbivory is known to induce accumulation of H_2_O_2_, which can play a role in defense [67], e.g. through direct toxicity [68], or indirectly by serving as a secondary signaling molecule in the induction of defense genes [69]. Enzymes that scavenge H_2_O_2_, e.g. catalases and peroxidases, can also be induced to higher levels upon attack by insect herbivores [70]. Ascorbate peroxidases reduce H_2_O_2_ to water [71], and can simultaneously oxidize phenolic compounds to quinones. This process, known as the browning reaction [72], is thought to inhibit insect feeding [73], [74]. Quinones can also cross-link with other compounds such as proteins [75], rendering them less digestible to insects [73]. Therefore, high levels of constitutive peroxidase activity in phloem tissue of Manchurian ash phloem may predispose it to respond more effectively when exposed to emerald ash borer attack.

Conversely, significant underexpression of the proteins listed in Table 3 in resistant Manchurian ash compared to susceptible North American ash species may contribute to susceptibility. However, based on the current literature, we cannot hypothesize what mechanisms these genes may govern that would result in enhanced susceptibility to the emerald ash borer, and therefore require further investigation.

**Table 3:** Phloem proteins putatively related to susceptibility identified in black, green, and white ash.

Protein Master Gel Number	Average Ratio (*P*-value)*			MASCOT Score [No. of peptides]	NCBI Accession Number‡	Protein ID	Annotation†
	B/M	G/M	W/M				
1962	15.13 (1.2E^−8^)	7.06 (1.3E^−7^)	5.34 (2.0E^−7^)	108 [3]	gi|147815877	Hypothetical protein [*Vitis vinifera*]	Protein Folding
430	20.36 (1.3E^−8^)	15.57 (1.5E^−9^)	11.53 (2.0E^−8^)	755 [14]	gi|147809607	Hypothetical protein [*Vitis vinifera*]	Proteolysis

*Proteins present in higher abundances (>5-fold) in black, green, and white ash relative to Manchurian ash.

† Annotations are Gene Ontology annotations for Biological Process.

‡ Searching NCBI Peptidome using the NCBI accession number of the matched protein will lead to detailed information about the peptides identified in this study. Black, green, and white ash peptide information can be obtained through Peptidome sample accession number PSM1313.

### Conclusions

Plants resist herbivores through complex combinations of constitutive and induced defenses [76]. This is the first study to identify constitutive proteins (a PR-10 protein, a phenyl-coumaran benzylic ether reductase, an aspartic protease, and a thylakoid-bound ascorbate peroxidase) that are strongly associated with the resistant Manchurian ash. Relative expression levels of the four proteins of interest from Manchurian ash show very little variation among individual biological replicates (Figure 4). Functional analysis of these genes will be the next step to fully characterize their potential role in resistance against the emerald ash borer. This can be achieved with two separate but complementary approaches. First, the exact genetic sequences must be identified through genomic or transcriptomic approaches coupled with information on protein sequence data obtained from this study. Functional characterization of these genes can then be accomplished by transforming susceptible ash species with the gene of interest. (transformation protocols are now available for green ash [77]). Second, development of an artificial diet for emerald ash borer [78] will provide a vehicle to test putative defense molecules, including the proteins identified in this study, directly against emerald ash borer larvae. Ultimately, discovery of genes associated with resistance to the emerald ash borer in coevolved Asian ash species will accelerate the development of resistant North American ash trees, similarly to the development of blight-resistant American chestnuts (*Castanea dentata* Marsh), which have been produced using both conventional breeding methods and transgenics [79]. Introgression of Asian resistance genes into susceptible North American ash species, either via hybridization or transgenics, will accelerate the generation of resistant genotypes for restoration of forested and urban ecosystems that have been severely impacted by the emerald ash borer invasion.

## Supporting Information

Figure S1 **A 1-D SDS-PAGE gel of protein extracts (20 µg per lane) from Manchurian, black, green, and white ash phloem tissues showing the high quality of the extracts.** Protein extracts are pools from eight biological replicates.
(TIF)

Figure S2 **A 2-D SDS-PAGE gel (p** ***I*** **3-10) of a pooled protein extract consisting of equal parts derived from 32 individual biological replicates (n = 8 each for Manchurian, black, green, and white ash).** Most of the proteins are found in the 4–7 p*I* range, which was subsequently used in all DIGE analyses.
(TIF)

Table S1 **Details of DIGE in-gel comparisons: M = Manchurian, G = green, W = white, B = black ash.** Numbers associated with each species represent biological replicates.
(DOC)

Table S2 **Proteins identified by MS and MASCOT analysis from Manchurian ash with an average ratio of 2 or greater when compared to black ash.**
(DOC)

Table S3 **Proteins identified by MS and MASCOT analysis from black ash with an average ratio of 2 or greater when compared to Manchurian ash.**
(DOC)
