# Supplementary figures and images for: Interspecific Proteomic Comparisons Reveal Ash Phloem Genes Potentially Involved in Constitutive Resistance to the Emerald Ash Borer
Source: PLoS One. 2011 Sep 15;6(9):e24863. doi: 10.1371/journal.pone.0024863 (PMC3174216; doi:10.1371/journal.pone.0024863)

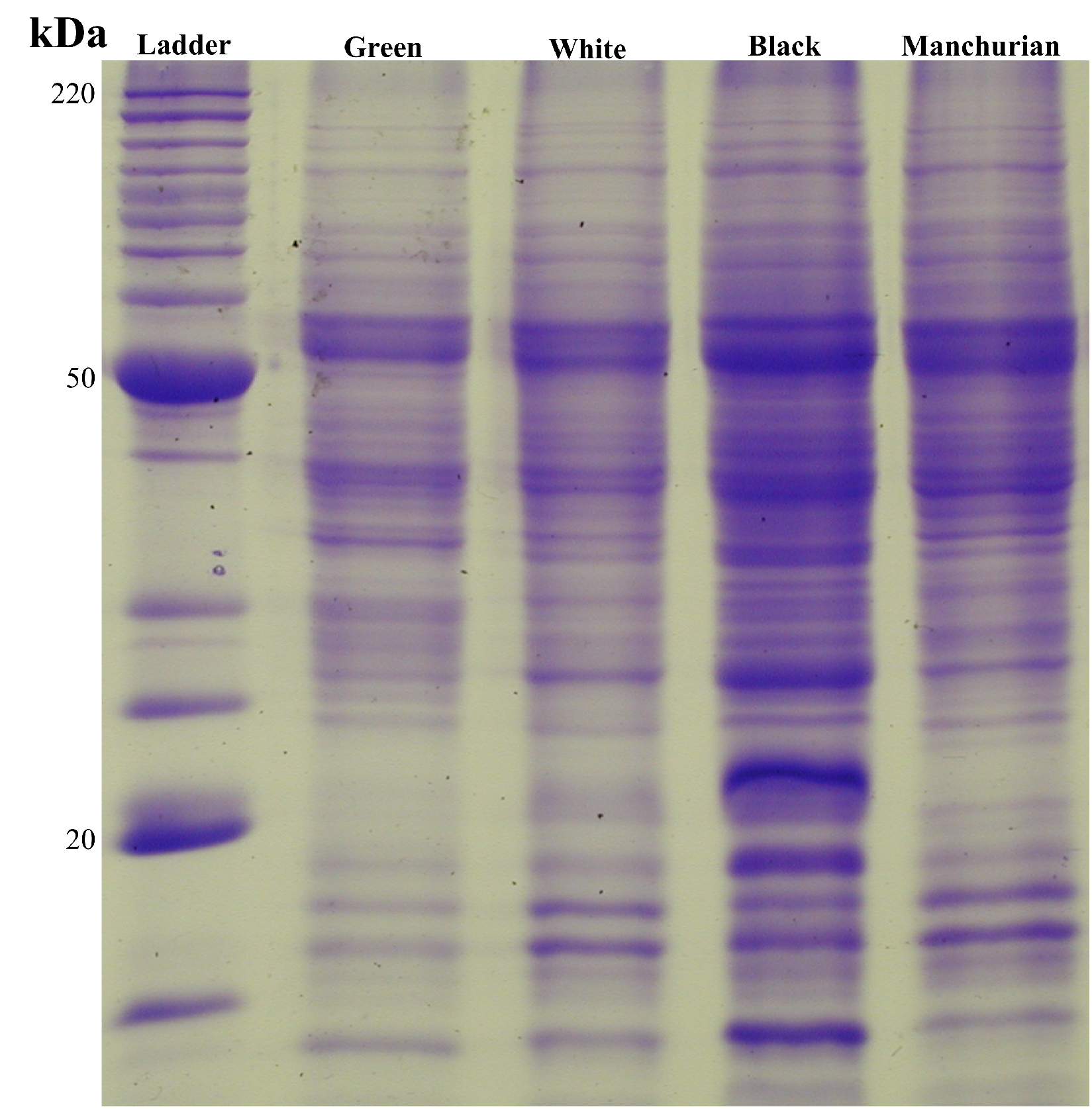

Supplement: Figure S1 — A 1-D SDS-PAGE gel of protein extracts (20 µg per lane) from Manchurian, black, green, and white ash phloem tissues showing the high quality of the extracts. Protein extracts are pools from eight biological replicates. (TIF) [file pone.0024863.s001.tif]

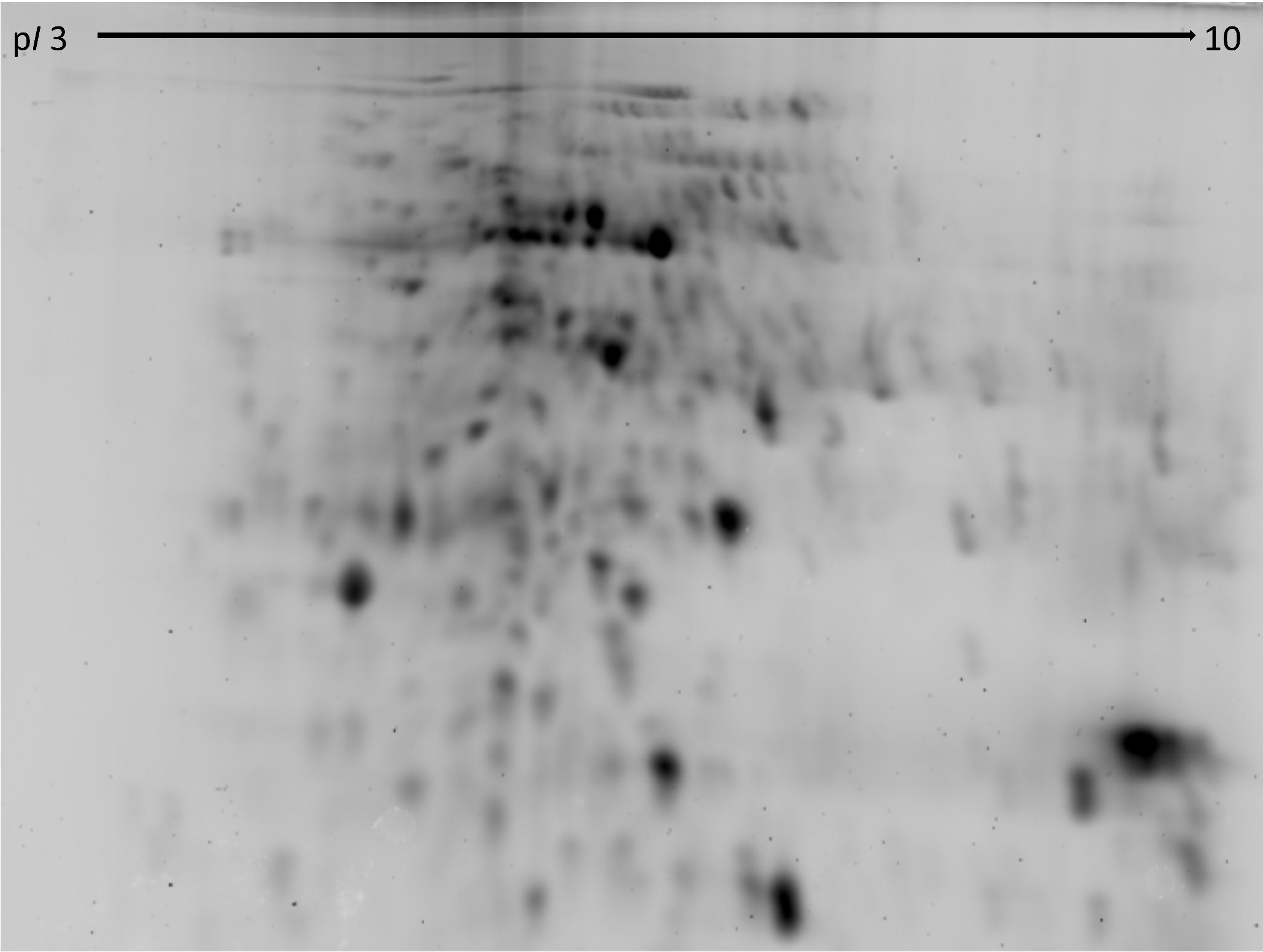

Supplement: Figure S2 — A 2-D SDS-PAGE gel (p I 3-10) of a pooled protein extract consisting of equal parts derived from 32 individual biological replicates (n = 8 each for Manchurian, black, green, and white ash). Most of the proteins are found in the 4–7 pI range, which was subsequently used in all DIGE analyses. (TIF) [file pone.0024863.s002.tif]
