# Supplementary material for: Interspecific Proteomic Comparisons Reveal Ash Phloem Genes Potentially Involved in Constitutive Resistance to the Emerald Ash Borer
Source: PLoS One. 2011 Sep 15;6(9):e24863. doi: 10.1371/journal.pone.0024863 (PMC3174216; doi:10.1371/journal.pone.0024863)
Supplement: Table S1 — Details of DIGE in-gel comparisons: M = Manchurian, G = green, W = white, B = black ash. Numbers associated with each species represent biological replicates. (DOC) [file pone.0024863.s003.doc]

Table S1. Details of DIGE in-gel comparisons: M = Manchurian, G = green, W = white, B = black ash. Numbers associated with each species represent biological replicates.

| **Gel Number** | **Cy3- labeled protein, 40 μg each** | **Cy5- labeled protein, 40 μg each** |
| --- | --- | --- |
|  |  |  |
| Gel 1 | M1 | W3 |
| Gel 2 | W1 | G3 |
| Gel 3 | G1 | B3 |
| Gel 4 | M2 | B4 |
| Gel 5 | B1 | M3 |
| Gel 6 | G2 | W4 |
| Gel 7 | B2 | G4 |
| Gel 8 | W2 | M4 |
| Gel 9 | W5 | B7 |
| Gel 10 | M5 | W7 |
| Gel 11 | B5 | W8 |
| Gel 12 | G5 | M7 |
| Gel 13 | W6 | G7 |
| Gel 14 | G6 | M8 |
| Gel 15 | B6 | G8 |
| Gel 16 | M6 | B8 |
