# Supplementary material for: Interspecific Proteomic Comparisons Reveal Ash Phloem Genes Potentially Involved in Constitutive Resistance to the Emerald Ash Borer
Source: PLoS One. 2011 Sep 15;6(9):e24863. doi: 10.1371/journal.pone.0024863 (PMC3174216; doi:10.1371/journal.pone.0024863)
Supplement: Table S2 — Proteins identified by MS and MASCOT analysis from Manchurian ash with an average ratio of 2 or greater when compared to black ash. (DOC) [file pone.0024863.s004.doc]

Table S2. Proteins identified by MS and MASCOT analysis from Manchurian ash with an average ratio of 2 or greater when compared to black, green, and white ash.

| **Protein Master Number^a^** | **NCBI Accession Number^b^** | **Protein name/Species^c^** | **Average Ratio (*P*-value)^d^** | | | **MASCOT Score^e^** |
| --- | --- | --- | --- | --- | --- | --- |
|  |  |  | **M/B** | **M/G** | **M/W** |  |
| **Biosynthetic Process^f^** | | |  |  |  |  |
| 686 | gi|121044462 | Granule-bound starch synthase I [*Lycium pumilum*] | 24.2 (2.10E-8) | 8.78 (4.8E-9) | 6.84 (3.5E-9) | 224 |
| ***Folic Acid and Derivative Biosynthetic Process*^g^** | | |  |  |  |  |
| 1381 | gi|157358403 | Unnamed protein product [*Vitis vinifera*] | 10.95 (4.60E-7) | 25.51 (2.6E-10) | 8.4 (4.4E-7) | 133 |
| ***Purine Ribonucleoside Salvage*** | | |  |  |  |  |
| 1291 | gi|147812626 | Hypothetical protein [*Vitis vinifera*] | 9.16 (5.70E-11) | 7.17 (1.6E-8) | 8.6 (1.8E-9) | 169 |
| **Carbohydrate Metabolic Process** | | |  |  |  |  |
| 322 | gi|157341193 | Unnamed protein product [*Vitis vinifera*] | 2.92 (1.20E-5) | 7.98 (5.6E-10) | 6.75 (7.2E-10) | 120 |
| ***Glycolysis*** |  |  |  |  |  |  |
| 769 | gi|3023685 | Enolase (*Alnus glutinosa*) | 2.97 (1.40E-5) | 4.77 (1.2E-8) | 3.44 (3.8E-7) | 742 |
| 787 | gi|3023685 | Enolase (*Alnus glutinosa*) | 12.05 (6.50E-11) | 33.21 (3.3E-13) | 11.56 (6.6E-12) | 908 |
| ***Mannose Metabolic Process*** | | |  |  |  |  |
| 326 | gi|157343878 | Unnamed protein product [*Vitis vinifera*] | 4.37 (2.60E-8) | 11.76 (2.4E-10) | 9.74 (2.0E-10) | 124 |
| ***Polysaccharide Catabolic Process*** | | |  |  |  |  |
| 662 | gi|147785379 | Hypothetical protein [*Vitis vinifera*] | 4.7(1.2E-4) | 6.73 (1.0E-6) | 4.65 (9.5E-5) | 263 |
| **Cellular Process** | | |  |  |  |  |
| ***Cell Redox Homeostasis*** | | |  |  |  |  |
| 440 | gi|225459587 | Hypothetical protein [*Vitis vinifera*] | 3.71 (1.40E-5) | 3.05 (1.1E-6) | 5.58 (3.7E-7) | 85 |
| 546 | gi|145666464 | Protein disulfide isomerase [*Zea mays*] | 4.72 (3.40E-9) | 6.02 (3.6E-11) | 5.77 (9.6E-11) | 268 |
| ***Malate Metabolic Process*** | | |  |  |  |  |
| 1345 | gi|126896 | Malate dehydrogenase | 4.25 (1.10E-5) | 2.64 (1.1E-6) | 3.52 (2.0E-8) | 347 |
| 434 | gi|228412 | Malic enzyme | 2.18 (5.9E-3) | 5.98 (6.7E-7) | 3.55 (3.3E-7) | 433 |
| 437 | gi|228412 | Malic enzyme | 4.91 (6.10E-8) | 5.13 (3.4E-7) | 4.37 (6.5E-7) | 366 |
| 444 | gi|228412 | Malic enzyme | 9.05 (8.70E-6) | 13.22 (1.6E-9) | 12.46 (1.5E-10) | 411 |
| **Cellular Amino Acid Metabolic Process** | | |  |  |  |  |
| ***L-Serine Biosynthetic Process*** | | |  |  |  |  |
| 536 | gi|15235282 | EDA9 (embryo sac development arrest  [*Arabidopsis thaliana*] | 2.16 (4.10E-8) | 4.9 (1.4E-11) | 5.63 (3.5E-9) | 169 |
| ***Methionine Biosynthetic Process*** | | |  |  |  |  |
| 958 | gi|118488207 | Unknown [*Populus trichocarpa*] | 5.62 (8.70E-11) | 2.04 (1.1E-7) | 2.64 (9.8E-9) | 74 |
| **Metabolic Process** | | |  |  |  |  |
| 1485 | gi|7578895 | Phenylcoumaran benzylic ether reductase homolog Fi1 [*Forsythia x intermedia*] | 4.78 (3.40E-9) | 8.6 (1.2E-8) | 4.38 (8.3E-8) | 148 |
| 1508 | gi|4731376 | Isoflavone reductase homolog Bet v 6.0101 [*Betula pendula*] | 7.82 (1.80E-5) | 2.51 (0.002) | 2.01 (0.01) | 145 |
| 1510 | gi|7578895 | Phenylcoumaran benzylic ether reductase homolog Fi1 [*Forsythia x intermedia*] | 33.57 (1.60E-9) | 25.6 (1.4E-9) | 27.44 (6.0E-9) | 435 |
| 1818 | gi|225440390 | Hypothetical protein [*Vitis vinifera*] | 12.29 (5.30E-9) | 4.86 (5.4E-9) | 3.85 (7.4E-8) | 323 |
| **Oxidation Reduction** | |  |  |  |  |  |
| 1045 | gi|71793966 | Alcohol dehydrogenase [*Alnus glutinosa*] | 2.31 (4.10E-5) | 3.6 (6.7E-9) | 4.2 (7.7E-9) | 142 |
| 1232 | gi|157352052 | Unnamed protein product [*Vitis vinifera*] | 2.31 (7.30E-5) | 3.34 (7.8E-7) | 5.23 (6.4E-7) | 96 |
| 991 | gi|117067068 | Monodehydroascorbate reductase [*Acanthus ebracteatus*] | 2.33 (9.90E-8) | 3.58 (2.6E-11) | 4.33 (2.5E-8) | 540 |
| **Photosynthesis** | |  |  |  |  |  |
| 1758 | gi|131385 | Oxygen-evolving enhancer protein 1 | 3.26 (8.70E-6) | 2.52 (2.4E-6) | 13.4 (1.6E-10) | 322 |
| ***Reductive Pentose-Phosphate Cycle*** | | |  |  |  |  |
| 1225 | gi|1173347 | Sedoheptulose-1,7-bisphosphatase, chloroplastic | 6.92 (1.70E-7) | 9.07 (8.2E-10) | 5.7 (4.2E-11) | 300 |
| **Protein Metabolic Process** | | |  |  |  |  |
| ***Proteolysis*** |  |  |  |  |  |  |
| 1537 | gi|13897888 | Putative aspartic protease [*Ipomoea batatas*] | 14.52 (6.50E-11) | 31.42 (1.3E-11) | 16.28 (6.3E-9) | 78 |
| **Response to Stress** | | |  |  |  |  |
| ***Defense Response*** | | |  |  |  |  |
| 2096 | gi|886683 | Major allergen [*Malus x domestica*] | 49.85 (7.10E-11) | 56.74 (2.1E-10) | 35.31 (3.0E-9) | 76 |
| 1317 | gi|2765081 | G5bf [*Arabidopsis thaliana*] | 2.45 (4.70E-6) | 3.72 (4.2E-8) | 2.7 (4.9E-7) | 70 |
| ***Hydrogen Peroxide Catabolic Process*** | | |  |  |  |  |
| 1627 | gi|25992557 | Thylakoid-bound ascorbate peroxidase [*Triticum aestivum*] | 10.15 (2.60E-9) | 10.03 (6.5E-11) | 9.18 (2.5E-9) | 110 |
| ***Response to Cold*** | | |  |  |  |  |
| 1409 | gi|157358403 | Unnamed protein product [*Vitis vinifera*] | 10.08 (1.30E-9) | 13.2 (9.3E-11) | 10.78 (4.7E-10) | 170 |
| 1469 | gi|5923684 | Putative 60S acidic ribosomal protein, 5' partial [*Arabidopsis thaliana*] | 8.61 (5.70E-11) | 9.84 (1.1E-7) | 26.76 (2.4E-10) | 135 |
| **Miscellaneous** | | |  |  |  |  |
| 502 | gi|157333098 | Unnamed protein product [*Vitis vinifera*] | 2.99 (3.70E-8) | 8.53 (4.9E-10) | 5.28 (1.8E-8) | 171 |
| 1481 | gi|2213425 | Hypothetical protein [*Citrus x paradisi*] | 6.67 (1.50E-10) | 5.98 (3.1E-10) | 6.89 (4.7E-9) | 127 |

a) Protein spot number matches the number to which the protein matches to the master gel spot map.

b) Accession number corresponds to the protein identification obtained through the MASCOT database search. Searching NCBI Peptidome using the NCBI accession number of the matched protein will lead to detailed information about the peptides identified in this study. Manchurian ash peptide information can be obtained through Peptidome sample accession number PSM1314.

c) Name of the protein identified through the MASCOT database search.

d) Average ratio of protein abundance for Manchurian (M)/black (B), M/green (G), and M/white (W) ash and *P*-value of the two-tailed Student’s t-test for each protein spot comparison between individual species comparisons.

e) MASCOT database score for peptide fragment matches to the database.

f) GOA (gene ontology annotation) parent class appears in boldface type for broad categorization of overall protein biological function.

g) GOA child terms appear in italics and refer to a specific biological function for certain proteins that also group under a specific parent term.
