# Supplementary material for: Interspecific Proteomic Comparisons Reveal Ash Phloem Genes Potentially Involved in Constitutive Resistance to the Emerald Ash Borer
Source: PLoS One. 2011 Sep 15;6(9):e24863. doi: 10.1371/journal.pone.0024863 (PMC3174216; doi:10.1371/journal.pone.0024863)
Supplement: Table S3 — Proteins identified by MS and MASCOT analysis from black ash with an average ratio of 2 or greater when compared to Manchurian ash. (DOC) [file pone.0024863.s005.doc]

Table S3. Proteins identified by MS and MASCOT analysis from black, green, and white ash with an average ratio of 2 or greater when compared to Manchurian ash.

| **Protein Master Number^a)^** | **NCBI Accession Number^b)^** | | **Protein name/Species^c)^** | **Average Ratio (*P-*value*)^d^*** | | | **MASCOT Score^e)^** |
| --- | --- | --- | --- | --- | --- | --- | --- |
|  |  |  |  | **B/M** | **G/M** | **W/M** |  |
|  | | | |  |  |  |  |
| **Carbohydrate Metabolic Process^f)^** | | | |  |  |  |  |
| ***Glycolysis*^g)^** |  | |  |  |  |  |  |
| 1176 | gi|1022803 | | Phosphoglycerate kinase [*Arabidopsis thaliana*] | 3.04 (7.40E-5) | 3.67 (1.1E-5) | 2.75 (3.9E-5) | 85 |
| 919 | gi|533474 | | 2-phospho-D-glycerate hydrolase | 2.15 (3.9E-4) | 12.85 (2.2E-9) | 2.13 (4.0E-4) | 164 |
| **Cellular Amino Acid Metabolic Process** | | | |  |  |  |  |
| ***Asparaginyl-tRNA Aminoacylation*** | | | |  |  |  |  |
| 386 | gi|15223302 | | ATP binding / aminoacyl-tRNA ligase [*Arabidopsis thaliana*] | 2.47 (1.4E-4) | 2.94 (3.1E-6) | 2.53 (1.0E-5) | 138 |
| **Cellular Process** | | |  |  |  |  |  |
| ***Malate Metabolic Process*** | | |  |  |  |  |  |
| 443 | | gi|1561774 | Malate dehydrogenase [*Vitis vinifera*] | 3.33 (9.60E-7) | 16.82 (3.3E-12) | 9.77 (1.5E-11) | 473 |
| **Metabolic Process** | | |  |  |  |  |  |
| 1871 | | gi|38112662 | Triose phosphate isomerase cytosolic isoform [*Solanum chacoense*] | 18.07 (5.70E-11) | 3.63 (2.4E-8) | 11.26 (3.1E-11) | 336 |
| **Oxidation Reduction** | | |  |  |  |  |  |
| 1572 | | gi|10334991 | NADPH-dependent mannose 6-phosphate reductase [*Orobanche ramosa*] | 2.47 (1.80E-5) | 7.82 (1.2E-11) | 2.81 (1.0E-6) | 171 |
|  | | |  |  |  |  |  |
| **Photosynthesis** | | |  |  |  |  |  |
| 1804 | | gi|21283 | Unnamed protein product [*Spinacia oleracea*] | 4.56 (1.40E-15) | 2.2 (5.8E-8) | 2.36 (1.5E-7) | 108 |
| 1687 | | gi|147791852 | Hypothetical protein [*Vitis vinifera*] | 4.02 (5.30E-9) | 52.53 (3.3E-13) | 4.48 (1.7E-7) | 496 |
| ***Reductive Pentose-Phosphate Cycle*** | | |  |  |  |  |  |
| 429 | gi|1750348 | | Ribulose-1,5-bisphosphate carboxylase/oxygenase large subunit [*Cosmelia rubra*] | 2.27 (2.80E-9) | 6.33 (4.3E-7) | 2.55 (6.5E-4) | 86 |
| ***Photosynthesis, Light Harvesting*** | | |  |  |  |  |  |
| 1992 | | gi|19184 | Type I (26 kD) CP29 polypeptide [*Solanum lycopersicum*] | 2.1 (6.80E-14) | 6.72 (5.4E-8) | 10.58 (4.7E-8) | 304 |
| 1914 | | gi|671737 | Chloropyll a/b binding protein [*Amaranthus hypochondriacus*] | 2.37 (1.10E-8) | 3.05 (1.2E-7) | 4.79 (1.1E-7) | 117 |
| **Protein Metabolic Process** | | | |  |  |  |  |
| ***Protein Folding*** | | |  |  |  |  |  |
| 1962 | gi|147815877 | | hypothetical protein [*Vitis vinifera*] | 15.13 (1.20E-8) | 7.06 (1.3E-7) | 5.34 (2.0E-7) | 108 |
| ***Proteolysis*** |  | |  |  |  |  |  |
| 360 | gi|147797811 | | Hypothetical protein [*Vitis vinifera*] | 4.32 (3.40E-12) | 4.32 (2.1E-6) | 4.61 (8.2E-7) | 597 |
| 363 | gi|147797811 | | Hypothetical protein [*Vitis vinifera*] | 3.33 (4.10E-11) | 2.22 (1.3E-4) | 3.56 (1.9E-6) | 445 |
| 430 | gi|147809607 | | Hypothetical protein [*Vitis vinifera*] | 20.36 (1.30E-8) | 15.57 (1.5E-9) | 11.53 (2.0E-8) | 755 |
| **Response to Stress** | | |  |  |  |  |  |
| ***Hydrogen Peroxide Catabolic Process*** | | | |  |  |  |  |
| 1935 | gi|15223049 | | APX1 (ascorbate peroxidase 1) [*Arabidopsis thaliana*] | 2.04 (3.9E-3) | 2.77 (2.3E-6) | 3.37 (2.6E-6) | 113 |
| **Miscellaneous** | | |  |  |  |  |  |
| 975 | gi|68565781 | | Ribulose bisphosphate carboxylase/oxygenase activase 2 | 10.2 (6.30E-6) | 2.17 (7.5E-3) | 2.66 (3.0E-3) | 646 |
| 1032 | gi|146432257 | | GDP-mannose-3',5'-epimerase [*Vitis vinifera*] | 2.16 (8.6E-4) | 2.15 (6.2E-6) | 2.12 (2.6E-5) | 435 |
| 1722 | gi|83283979 | | Protein transport SEC13-like protein [*Solanum tuberosum*] | 2.95 (1.80E-6) | 4.4 (8.1E-9) | 3.92 (2.7E-8) | 107 |

a) Protein spot number matches the number to where the protein is located on the master gel spot map.

b) Accession number corresponds to the protein identification obtained through the MASCOT database search. Searching NCBI Peptidome using the NCBI accession number of the matched protein will lead to detailed information about the peptides identified in this study. Peptide information can be obtained through Peptidome sample accession number PSM1313.

c) Name of the protein identified through the MASCOT database search.

d) Average ratio of protein abundance for black (B)/Manchurian (M), green (G)/M, and white (W)/M ash and *P*-value of the two-tailed Student’s t-test for each protein spot for individual comparisons against Manchurian ash.

e) MASCOT database score for peptide fragment matches to the database.

f) GOA (gene ontology annotation) parent class appears in boldface type for broad categorization of overall protein biological function.

g) GOA child terms appear in italics and refer to a specific biological function for certain proteins that also group under a specific parent term.
